# Supplementary material for: Psychometric validation of the Hospital Stress Questionnaire
Source: PLoS One. 2025 Apr 9;20(4):e0321188. doi: 10.1371/journal.pone.0321188 (PMC11981188; doi:10.1371/journal.pone.0321188)
Supplement: S1 File — (DOCX) [file pone.0321188.s001.docx]

Appendices

**Appendix S1.** Hospital experiences survey

[Information sheet and Consent form presented]

Are you over 18 years old? [can only proceed if answered “yes”]

Have you stayed in a hospital in the UK? [can only proceed if answered “yes”]

Was that stay in hospital longer than 24 hours? [can only proceed if answered “yes”]

Was that stay in hospital in the past 12 months? [can only proceed if answered “yes”]

Was that stay in hospital for psychiatric or maternity care? [can only proceed if answered “no”]

Thank you for taking the time to complete this survey. You have been invited to take part because you are (i) over 18 years old and (ii) have stayed in a UK hospital (iii) for at least 24 hours (iv) in the last 12 months, (v) for any reason that was NOT for psychiatric or maternity care. We are interested in hearing about your hospital experience.

In this survey, you will be asked to complete some background information about yourself and your hospital stay. This will be followed by three questionnaires on your hospital experience, stress levels, and overall health. Altogether, the survey should take around 15 minutes to complete. For all questions, the best approach is to answer quickly.

What is your current age?

What age were you when you went into hospital?

What is your sex?

Is the gender you identify with the same as your sex registered at birth?

What is your ethnic group?

What is your highest level of education completed?

What is your legal marital/civil partnership status?

In your life, approximately how many times have you stayed in hospital for more than 24 hours?

In the past 12 months, how many times have you stayed in hospital for more than 24 hours?

When was the most recent time you stayed in hospital for over 24 hours? (Please give the exact date, if possible)

**IMPORTANT**
For the rest of the questions, please answer with your **most recent hospital stay** in mind (i.e. your answer from the previous question).

How long were you in hospital?

What was the reason for your hospital stay? (e.g. 'I had a heart attack'). If unsure, please write 'I don’t know'.

Which UK hospital did you stay in? (e.g. 'Leeds General Infirmary').

What type of ward did you stay on? (e.g. 'cardiology' or 'intensive care unit'). If unsure, please write 'I don’t know'.

Did you have surgery?

Was your hospital stay planned beforehand?

What type of hospital did you stay in?

How prepared did you feel to go home when you left hospital? [1–10]

How long after leaving hospital did it take you to get back to the usual activities you did before going into hospital? (e.g., driving, work, cooking, housework, leisure, etc.)

Was this hospital stay a readmission from a previous hospital stay?

If yes, when was the original hospital stay? (Please give the exact date, if possible)

Did you experience any of the following within approximately 6 weeks of leaving hospital:

- Did you have any sores or wounds that would not heal?
- Did you have any infections?
- Did you have a fall?
- Did you have any additional problems that lead to contacting the GP, A&E, or anyone else?

In the six weeks after leaving hospital, how vulnerable did you feel? E.g., feeling weak, unsafe, or that your health might get worse. [1–10]

In the weeks leading up to your hospital stay…

- How often did you feel that you lacked companionship?
- How often did you feel left out?
- How often did you feel isolated from others?

In the month leading up to your hospital stay…

- [Concerning the people you are related to:]
- How many relatives did you see or hear from at least once in the month?
- How many relatives did you feel close to such that you could call on them for help?
- How many relatives did you feel at ease with such that you could talk about private matters?
- [Considering all of your friends and neighbours:]
- How many of your friends did you see or hear from at least once in the month?
- How many friends did you feel close to such that you could call on them for help?
- How many friends did you feel at ease with such that you could talk about private matters?

**Questionnaire (1 of 3)**
**IMPORTANT INFORMATION IN BOLD!**

**What is the questionnaire about?**
This questionnaire aims to measure how much stress you experienced **during your hospital stay**. While answering the questions, consider 'stress' as feeling tense, worried, or wound up by a situation.

**Completing the questionnaire**
Please read each question carefully, keeping in mind your **most recent** stay in hospital and select one option for each question. **If you did not experience any of the events described in the questions**, please select N/A. This should take you around 5-10 minutes to complete. The best approach is to answer quickly.

| During your hospital stay, please rate how much stress you felt as a result of: | | | | | | | | | | | | |
| --- | --- | --- | --- | --- | --- | --- | --- | --- | --- | --- | --- | --- |
|  | Not at all stressful  (1) | | | | | | Extremely stressful  (10) | | | | |  |
| 1. Not sleeping well | 1 | 2 | 3 | 4 | 5 | 6 | | 7 | 8 | 9 | 10 | N/A |
| 2. Feeling helpless or not in control | 1 | 2 | 3 | 4 | 5 | 6 | | 7 | 8 | 9 | 10 | N/A |
| 3. Having pain or discomfort from your treatment | 1 | 2 | 3 | 4 | 5 | 6 | | 7 | 8 | 9 | 10 | N/A |
| 4. Staying in a noisy room | 1 | 2 | 3 | 4 | 5 | 6 | | 7 | 8 | 9 | 10 | N/A |
| 5. The staff not communicating well with you | 1 | 2 | 3 | 4 | 5 | 6 | | 7 | 8 | 9 | 10 | N/A |
| 6. The staff being too busy | 1 | 2 | 3 | 4 | 5 | 6 | | 7 | 8 | 9 | 10 | N/A |
| 7. The staff not being caring or friendly | 1 | 2 | 3 | 4 | 5 | 6 | | 7 | 8 | 9 | 10 | N/A |
| 8. Having to wait a lot | 1 | 2 | 3 | 4 | 5 | 6 | | 7 | 8 | 9 | 10 | N/A |
| 9. The food being bad or not meeting your dietary requirements | 1 | 2 | 3 | 4 | 5 | 6 | | 7 | 8 | 9 | 10 | N/A |
| 10. Feeling like you could not leave your bed or ward | 1 | 2 | 3 | 4 | 5 | 6 | | 7 | 8 | 9 | 10 | N/A |
| 11. Not knowing what was going to happen to you | 1 | 2 | 3 | 4 | 5 | 6 | | 7 | 8 | 9 | 10 | N/A |
| 12. Feeling bored | 1 | 2 | 3 | 4 | 5 | 6 | | 7 | 8 | 9 | 10 | N/A |
| 13. The staff making a mistake that caused you harm | 1 | 2 | 3 | 4 | 5 | 6 | | 7 | 8 | 9 | 10 | N/A |
| 14. Worrying that your treatment/medication will have side effects | 1 | 2 | 3 | 4 | 5 | 6 | | 7 | 8 | 9 | 10 | N/A |
| 15. Fearing your health will get worse | 1 | 2 | 3 | 4 | 5 | 6 | | 7 | 8 | 9 | 10 | N/A |
| 16. Feeling like the staff were not listening to you | 1 | 2 | 3 | 4 | 5 | 6 | | 7 | 8 | 9 | 10 | N/A |
| Please select ‘7’ to show you are paying attention | 1 | 2 | 3 | 4 | 5 | 6 | | 7 | 8 | 9 | 10 | N/A |
| 17. The other patients being difficult | 1 | 2 | 3 | 4 | 5 | 6 | | 7 | 8 | 9 | 10 | N/A |
| 18. Feeling lonely | 1 | 2 | 3 | 4 | 5 | 6 | | 7 | 8 | 9 | 10 | N/A |
| 19. Missing loved ones | 1 | 2 | 3 | 4 | 5 | 6 | | 7 | 8 | 9 | 10 | N/A |
| 20. The staff being rude or unprofessional | 1 | 2 | 3 | 4 | 5 | 6 | | 7 | 8 | 9 | 10 | N/A |
| 21. The staff not being responsive to the buzzer | 1 | 2 | 3 | 4 | 5 | 6 | | 7 | 8 | 9 | 10 | N/A |
| 22. Having blood taken | 1 | 2 | 3 | 4 | 5 | 6 | | 7 | 8 | 9 | 10 | N/A |
| 23. Being disturbed by observations (e.g. blood pressure) | 1 | 2 | 3 | 4 | 5 | 6 | | 7 | 8 | 9 | 10 | N/A |
| 24. Having to rely on others | 1 | 2 | 3 | 4 | 5 | 6 | | 7 | 8 | 9 | 10 | N/A |
| 25. Not feeling safe | 1 | 2 | 3 | 4 | 5 | 6 | | 7 | 8 | 9 | 10 | N/A |
| 26. Worrying about the wellbeing of other patients | 1 | 2 | 3 | 4 | 5 | 6 | | 7 | 8 | 9 | 10 | N/A |
| 27. The hospital not being organised | 1 | 2 | 3 | 4 | 5 | 6 | | 7 | 8 | 9 | 10 | N/A |
| 28. Sharing a room with strangers | 1 | 2 | 3 | 4 | 5 | 6 | | 7 | 8 | 9 | 10 | N/A |
| 29. The staff not communicating well with each other | 1 | 2 | 3 | 4 | 5 | 6 | | 7 | 8 | 9 | 10 | N/A |
| Please select ‘4’ to show you are paying attention | 1 | 2 | 3 | 4 | 5 | 6 | | 7 | 8 | 9 | 10 | N/A |
| 30. Not being allowed access to your usual medication | 1 | 2 | 3 | 4 | 5 | 6 | | 7 | 8 | 9 | 10 | N/A |
| 31. Hearing or seeing emergencies | 1 | 2 | 3 | 4 | 5 | 6 | | 7 | 8 | 9 | 10 | N/A |
| 32. Feeling homesick | 1 | 2 | 3 | 4 | 5 | 6 | | 7 | 8 | 9 | 10 | N/A |
| 33. Not getting enough to drink | 1 | 2 | 3 | 4 | 5 | 6 | | 7 | 8 | 9 | 10 | N/A |
| 34. Being in an overcrowded ward | 1 | 2 | 3 | 4 | 5 | 6 | | 7 | 8 | 9 | 10 | N/A |
| 35. Overhearing the staff having conversations | 1 | 2 | 3 | 4 | 5 | 6 | | 7 | 8 | 9 | 10 | N/A |
| 36. Medical procedure getting cancelled or delayed | 1 | 2 | 3 | 4 | 5 | 6 | | 7 | 8 | 9 | 10 | N/A |
| 37. Not being involved in the treatment plan | 1 | 2 | 3 | 4 | 5 | 6 | | 7 | 8 | 9 | 10 | N/A |
| 38. Equipment or supplies lacking | 1 | 2 | 3 | 4 | 5 | 6 | | 7 | 8 | 9 | 10 | N/A |
| 39. Having to deal with the symptoms of your illness (e.g. sickness) | 1 | 2 | 3 | 4 | 5 | 6 | | 7 | 8 | 9 | 10 | N/A |
| 40. Being in a room that was too hot or too cold | 1 | 2 | 3 | 4 | 5 | 6 | | 7 | 8 | 9 | 10 | N/A |
| 41. Being in an unfamiliar place | 1 | 2 | 3 | 4 | 5 | 6 | | 7 | 8 | 9 | 10 | N/A |
| 42. Being in an unclean room | 1 | 2 | 3 | 4 | 5 | 6 | | 7 | 8 | 9 | 10 | N/A |
| 43. Worrying about loved ones | 1 | 2 | 3 | 4 | 5 | 6 | | 7 | 8 | 9 | 10 | N/A |
| 44. Having to follow the hospital’s schedule | 1 | 2 | 3 | 4 | 5 | 6 | | 7 | 8 | 9 | 10 | N/A |
| 45. Being in a room that was too bright or has no natural light | 1 | 2 | 3 | 4 | 5 | 6 | | 7 | 8 | 9 | 10 | N/A |
| 46. Having tubes in your nose, mouth, or other body parts | 1 | 2 | 3 | 4 | 5 | 6 | | 7 | 8 | 9 | 10 | N/A |
| 47. Being reminded of loved ones who passed away while in hospital | 1 | 2 | 3 | 4 | 5 | 6 | | 7 | 8 | 9 | 10 | N/A |
| Please select ‘10’ to show you are paying attention | 1 | 2 | 3 | 4 | 5 | 6 | | 7 | 8 | 9 | 10 | N/A |
| 48. The staff not asking for consent before treating you | 1 | 2 | 3 | 4 | 5 | 6 | | 7 | 8 | 9 | 10 | N/A |
| 49. Not being able to do your usual activities | 1 | 2 | 3 | 4 | 5 | 6 | | 7 | 8 | 9 | 10 | N/A |
| 50. Fearing that you may pick up an illness from being in hospital | 1 | 2 | 3 | 4 | 5 | 6 | | 7 | 8 | 9 | 10 | N/A |
| 51. Feeling like you had no privacy | 1 | 2 | 3 | 4 | 5 | 6 | | 7 | 8 | 9 | 10 | N/A |
| 52. Feeling like you were not being treated like a person | 1 | 2 | 3 | 4 | 5 | 6 | | 7 | 8 | 9 | 10 | N/A |
| 53. Worrying about money | 1 | 2 | 3 | 4 | 5 | 6 | | 7 | 8 | 9 | 10 | N/A |
| 54. Feeling like your life was on hold or you were missing out | 1 | 2 | 3 | 4 | 5 | 6 | | 7 | 8 | 9 | 10 | N/A |
| 55. Having poor Wi-Fi or phone signal | 1 | 2 | 3 | 4 | 5 | 6 | | 7 | 8 | 9 | 10 | N/A |
| 56. Not being sure of your diagnosis | 1 | 2 | 3 | 4 | 5 | 6 | | 7 | 8 | 9 | 10 | N/A |
| 57. Worrying how you will cope once leaving hospital | 1 | 2 | 3 | 4 | 5 | 6 | | 7 | 8 | 9 | 10 | N/A |
| 58. Missing your usual small comforts (e.g. hot tea) | 1 | 2 | 3 | 4 | 5 | 6 | | 7 | 8 | 9 | 10 | N/A |
| 59. Not being able to pray or do other religious activities | 1 | 2 | 3 | 4 | 5 | 6 | | 7 | 8 | 9 | 10 | N/A |
| 60. Feeling like the staff focused on other patients more than you | 1 | 2 | 3 | 4 | 5 | 6 | | 7 | 8 | 9 | 10 | N/A |
| 61. Not knowing the hospital rules | 1 | 2 | 3 | 4 | 5 | 6 | | 7 | 8 | 9 | 10 | N/A |
| 62. Having to wear a hospital gown | 1 | 2 | 3 | 4 | 5 | 6 | | 7 | 8 | 9 | 10 | N/A |
| 63. Needing help going to the bathroom | 1 | 2 | 3 | 4 | 5 | 6 | | 7 | 8 | 9 | 10 | N/A |
| 64. Worrying that your appearance might change (e.g. scars) | 1 | 2 | 3 | 4 | 5 | 6 | | 7 | 8 | 9 | 10 | N/A |
| 65. Being transferred between wards or hospitals | 1 | 2 | 3 | 4 | 5 | 6 | | 7 | 8 | 9 | 10 | N/A |
| 66. The hospital not meeting your individual needs (e.g. disability) | 1 | 2 | 3 | 4 | 5 | 6 | | 7 | 8 | 9 | 10 | N/A |
| 67. Not being able to smoke, drink alcohol, or use other substances | 1 | 2 | 3 | 4 | 5 | 6 | | 7 | 8 | 9 | 10 | N/A |
| Other (write in) | 1 | 2 | 3 | 4 | 5 | 6 | | 7 | 8 | 9 | 10 | N/A |

Overall, how stressed did you feel during your hospital stay? [1–10]

If you have any additional comments, please write them here: ________

**Questionnaire (2 of 3)**
The questions in this scale ask you about your feelings and thoughts **during your hospital stay**. In each case, you will be asked to indicate by circling how often you felt or thought a certain way.

[PSS-10 questions, each asking “While in hospital…”]

**Questionnaire (3 of 3)**
Under each heading, please select the **ONE** box that best describes your health **IN THE TWO WEEKS AFTER BEING DISCHARGED FROM HOSPITAL.**

[EQ-5D-5L questions]

We would like to know how good or bad your health was **in the two weeks after being discharged from hospital.**
This scale is numbered from 0 to 100.
 • 100 means the best health you can imagine.
 • 0 means the worst health you can imagine.

[EQ VAS]

**Appendix S2.** Sample demographics

Table S1. More detailed breakdown of ethnicities within current sample.

| **Ethnicity** | **N** | **%** |
| --- | --- | --- |
| **Asian or Asian British** | **55** | **8.3** |
| Indian | 19 | 2.9 |
| Pakistani | 15 | 2.3 |
| Bangladeshi | 3 | 0.5 |
| Chinese | 8 | 1.2 |
| Other | 10 | 1.5 |
| **Black, Black British, Caribbean or African** | **30** | **4.5** |
| Caribbean | 8 | 1.2 |
| African | 22 | 3.3 |
| Other | 0 | 0 |
| **Mixed or Multiple ethnic groups** | **20** | **3.0** |
| White and Black Caribbean | 13 | 2.0 |
| White and Black African | 1 | 0.2 |
| White and Asian | 3 | 0.5 |
| Other | 3 | 0.5 |
| **White** | **548** | **83.0** |
| English, Welsh, Scottish, Northern Irish, or British | 518 | 78.5 |
| Irish | 5 | 0.8 |
| Gypsy or Irish Traveller | 0 | 0 |
| Roma | 0 | 0 |
| Other | 25 | 3.8 |
| **Other ethnic group** | **6** | **0.9** |
| Arab | 2 | 0.3 |
| Other | 4 | 0.6 |
| **Prefer not to say** | **1** | **0.2** |
| **Total** | **660** | **100** |

**Gender same as sex at birth:**

Yes – 657 (99.5%)

No – 0 (0%)

Prefer not to say – 3 (0.5%)

**Education:**

None – 23 (3.5%)

GCSE (or equivalent) – 128 (19.4%)

A-Level (or equivalent) – 154 (23.3%)

Undergraduate – 237 (35.9%)

Postgraduate – 118 (17.9%)

**Marital:**

Single – 172 (26.1%)

Married – 368 (55.8%)

In a registered civil partnership – 15 (2.3%)

Separated, but still legally married / in a civil partnership – 12 (1.8%)

Divorced/Dissolved – 49 (7.4%)

Widowed – 40 (6.1%)

Prefer not to say – 4 (0.6%)

**Surgery:**

Yes – 276 (41.8%)

No – 380 (57.6%)

I’m not sure – 4 (0.6%)

**Planned/unplanned:**

Planned – 223 (33.8%)

Emergency – 437 (66.2%)

**Appendix S3.** Mean and standard deviation of each item

Table S2. Mean and standard deviation of each item – ranked from most to least stressful.

| **Rank** | **HSQ Item** | **Mean** | **SD** |
| --- | --- | --- | --- |
| 1 | 1. Not sleeping well | 5.82 | 2.80 |
| 2 | 2. Feeling helpless or not in control | 5.67 | 2.85 |
| 3 | 19. Missing loved ones | 5.63 | 3.06 |
| 4 | 12. Feeling bored | 5.56 | 2.90 |
| 5 | 3. Having pain or discomfort from your treatment | 5.54 | 2.73 |
| 6 | 8. Having to wait a lot | 5.52 | 2.92 |
| 7 | 4. Staying in a noisy room | 5.24 | 3.04 |
| 8 | 49. Not being able to do your usual activities | 5.24 | 2.94 |
| 9 | 11. Not knowing what was going to happen to you | 5.22 | 3.08 |
| 10 | 24. Having to rely on others | 5.03 | 2.97 |
| 11 | 15. Fearing your health will get worse | 5.00 | 3.15 |
| 12 | 39. Having to deal with the symptoms of your illness (e.g. sickness) | 4.97 | 3.07 |
| 13 | 51. Feeling like you had no privacy | 4.87 | 3.19 |
| 14 | 6. The staff being too busy | 4.80 | 2.95 |
| 15 | 50. Fearing that you may pick up an illness from being in hospital | 4.80 | 3.13 |
| 16 | 58. Missing your usual small comforts (e.g. hot tea) | 4.72 | 3.04 |
| 17 | 32. Feeling homesick | 4.68 | 3.17 |
| 18 | 10. Feeling like you could not leave your bed or ward | 4.46 | 2.94 |
| 19 | 28. Sharing a room with strangers | 4.43 | 3.15 |
| 20 | 41. Being in an unfamiliar place | 4.38 | 2.98 |
| 21 | 43. Worrying about loved ones | 4.32 | 3.01 |
| 22 | 56. Not being sure of your diagnosis | 4.30 | 3.20 |
| 23 | 57. Worrying how you will cope once leaving hospital | 4.30 | 3.00 |
| 24 | 5. The staff not communicating well with you | 4.26 | 2.90 |
| 25 | 18. Feeling lonely | 4.25 | 2.86 |
| 26 | 9. The food being bad or not meeting your dietary requirements | 4.23 | 2.90 |
| 27 | 54. Feeling like your life was on hold or you were missing out | 4.17 | 2.99 |
| 28 | 14. Worrying that your treatment/medication will have side effects | 4.03 | 2.99 |
| 29 | 55. Having poor Wi-Fi or phone signal | 3.75 | 3.07 |
| 30 | 40. Being in a room that was too hot or too cold | 3.73 | 2.92 |
| 31 | 23. Being disturbed by observations (e.g. blood pressure) | 3.68 | 2.74 |
| 32 | 44. Having to follow the hospital’s schedule | 3.66 | 2.77 |
| 33 | 17. The other patients being difficult | 3.61 | 2.73 |
| 34 | 22. Having blood taken | 3.55 | 2.78 |
| 35 | 46. Having tubes in your nose, mouth, or other body parts | 3.49 | 3.00 |
| 36 | 34. Being in an overcrowded ward | 3.45 | 2.85 |
| 37 | 45. Being in a room that was too bright or has no natural light | 3.41 | 2.86 |
| 38 | 63. Needing help going to the bathroom | 3.41 | 2.93 |
| 39 | 16. Feeling like the staff were not listening to you | 3.33 | 2.76 |
| 40 | 27. The hospital not being organised | 3.32 | 2.67 |
| 41 | 62. Having to wear a hospital gown | 3.32 | 2.74 |
| 42 | 7. The staff not being caring or friendly | 3.28 | 2.58 |
| 43 | 29. The staff not communicating well with each other | 3.25 | 2.70 |
| 44 | 35. Overhearing the staff having conversations | 3.10 | 2.57 |
| 45 | 37. Not being involved in the treatment plan | 3.10 | 2.67 |
| 46 | 36. Medical procedure getting cancelled or delayed | 3.06 | 2.87 |
| 47 | 26. Worrying about the wellbeing of other patients | 2.95 | 2.35 |
| 48 | 31. Hearing or seeing emergencies | 2.92 | 2.55 |
| 49 | 52. Feeling like you were not being treated like a person | 2.92 | 2.58 |
| 50 | 21. The staff not being responsive to the buzzer | 2.89 | 2.59 |
| 51 | 53. Worrying about money | 2.82 | 2.68 |
| 52 | 25. Not feeling safe | 2.80 | 2.44 |
| 53 | 47. Being reminded of loved ones who passed away while in hospital | 2.80 | 2.72 |
| 54 | 33. Not getting enough to drink | 2.78 | 2.39 |
| 55 | 64. Worrying that your appearance might change (e.g. scars) | 2.74 | 2.57 |
| 56 | 65. Being transferred between wards or hospitals | 2.70 | 2.58 |
| 57 | 42. Being in an unclean room | 2.57 | 2.39 |
| 58 | 38. Equipment or supplies lacking | 2.52 | 2.36 |
| 59 | 61. Not knowing the hospital rules | 2.49 | 2.19 |
| 60 | 60. Feeling like the staff focused on other patients more than you | 2.48 | 2.23 |
| 61 | 20. The staff being rude or unprofessional | 2.40 | 2.27 |
| 62 | 13. The staff making a mistake that caused you harm | 2.32 | 2.43 |
| 63 | 30. Not being allowed access to your usual medication | 2.31 | 2.27 |
| 64 | 48. The staff not asking for consent before treating you | 2.22 | 2.10 |
| 65 | 66. The hospital not meeting your individual needs (e.g. disability) | 2.09 | 2.15 |
| 66 | 67. Not being able to smoke, drink alcohol, or use other substances | 1.95 | 2.16 |
| 67 | 59. Not being able to pray or do other religious activities | 1.75 | 1.82 |
